# Gfa1 (glutamine fructose-6-phosphate aminotransferase) is essential for *Aspergillus fumigatus *growth and virulence

**DOI:** 10.1186/s12915-025-02184-0

**Published:** 2025-03-13

**Authors:** Qijian Qin, Pingzhen Wei, Sayed Usman, Chukwuemeka Samson Ahamefule, Cheng Jin, Bin Wang, Kaizhou Yan, Daan M. F. van Aalten, Wenxia Fang

**Affiliations:** 1 Institute of Biological Sciences and Technology, Guangxi Academy of Sciences, Nanning, 530007 China; 2 State Key Laboratory of Mycology, Institute of Microbiology, Chinese Academy of Sciences, Beijing, 100101 China; 3 School of Life Sciences, University of Dundee, Dundee, UK; 4 Section of Neurobiology and DANDRITE, Department of Molecular Biology and Genetics, Aarhus University, Aarhus, Denmark

**Keywords:** *Aspergillus fumigatus*, Cell wall, Glutamine fructose-6-phosphate amidotransferase, Drug target

## Abstract

**Background:**

*Aspergillus fumigatus*, the primary etiological agent of invasive aspergillosis, causes over 1.8 million deaths annually. Targeting cell wall biosynthetic pathways offers a promising antifungal strategy. Gfa1, a rate-limiting enzyme in UDP-GlcNAc synthesis, plays a pivotal role in the hexosamine biosynthetic pathway (HBP).

**Results:**

Deletion of *gfa1* (Δ*gfa1*) results in auxotrophy for glucosamine (GlcN) or N-acetylglucosamine (GlcNAc). Under full recovery (FR) conditions, where minimal medium is supplemented with 5 mM GlcN as the sole carbon source, the Δ*gfa1* mutant shows growth comparable to the wild-type (WT). However, when supplemented with 5 mM GlcN and 55 mM glucose, growth is partially repressed, likely due to carbon catabolite repression, a condition termed partial repression (PR). Under PR conditions, Δ*gfa1* exhibits compromised growth, reduced conidiation, defective germination, impaired cell wall integrity, and increased sensitivity to endoplasmic reticulum (ER) stress and high temperatures. Additionally, Δ*gfa1* demonstrates disruptions in protein homeostasis and iron metabolism. Transcriptomic analysis of the mutant under PR conditions reveals significant alterations in carbohydrate and amino acid metabolism, unfolded protein response (UPR) processes, and iron assimilation. Importantly, Gfa1 is essential for *A. fumigatus* virulence, as demonstrated in *Caenorhabditis elegans* and *Galleria mellonella* infection models.

**Conclusions:**

These findings underscore the critical role of Gfa1 in fungal pathogenicity and suggest its potential as a therapeutic target for combating *A. fumigatus* infections.

**Supplementary Information:**

The online version contains supplementary material available at 10.1186/s12915-025-02184-0.

## Importance

Identifying new drug targets is essential for advancing antifungal therapies and combating critical fungal pathogens. This study addresses this urgent need by investigating the role of Gfa1 through the creation of a *gfa1* deletion mutant, complemented with glucosamine or N-acetylglucosamine. Our findings highlight the multifaceted functions of Gfa1 in cell survival and development, including its crucial roles in maintaining protein homeostasis, regulating iron metabolism, and driving fungal virulence. These insights provide a deeper understanding of *A. fumigatus* pathogenesis and underscore Gfa1’s potential as a promising target for antifungal drug development.

## Background

*Aspergillus fumigatus* is responsible for a spectrum of diseases collectively known as aspergillosis, ranging from localized infections to severe allergic bronchopulmonary aspergillosis, and potentially life-threatening invasive aspergillosis (IA) [1]. IA affects approximately 2.1 million people globally each year, resulting in over 1.8 million deaths annually [2]. The occurrence of COVID-19-associated invasive pulmonary aspergillosis (CAPA) has raised concerns about this superinfection becoming a new cause of mortality [3]. Recently, the World Health Organization (WHO) identified *A. fumigatus* as a critical priority pathogen in their WHO fungal priority pathogens list (FPPL), underscoring its impact on human health and the threat posed by resistance emergence [4]. With clinical resistance to existing antifungals on the rise, the situation has become increasingly alarming [5]. This highlights the urgent need to develop novel antifungal strategies and targets to combat treatment failures and effectively manage aspergillosis.

The fungal cell wall plays a crucial role in determining cell morphology and providing structural integrity. It serves as a barrier, offering protection against various environmental stressors such as UV radiation, temperature, desiccation, and enzymatic attacks, while also facilitating adherence to diverse substrates [6]. Receptors embedded within the cell wall enable the fungus to perceive and respond to a broad range of environmental cues, activating cell signaling pathways in the process [7]. Notably, the composition of the fungal cell wall is species-specific, with filamentous fungi primarily consisting of glucan, chitin, and glycoproteins. These components collectively account for significant proportions of the cell wall dry weight, with glucan comprising 50–60%, chitin 10–20%, and glycoproteins 20–30% [8]. Importantly, many of the chemical constituents of the fungal cell wall are absent in the human body, rendering them possible targets for the development of clinical antifungal drugs and immunotherapies [9].

Gfa1 is the first and rate limiting enzyme[10] in the HBP. In lower and higher eukaryotes, the HBP pathway metabolizes fructose-6-phosphate (Fru6P) to UDP-GlcNAc and is universally conserved. UDP-GlcNAc is an essential amino sugar donor for cell wall chitin biosynthesis [11], and also involved in protein glycosylation, and biosynthesis of lipids and other important biomolecules [12]. The functional significance of Gfa1 has been studied in a range of organisms. Gfa1 plays a significant role in the regulatory mechanism implicated in compensatory responses to cell wall damage. In *Saccharomyces cerevisiae*, it has been reported that Gfa1 is responsible for triggering the activation of the chitin biosynthesis pathway, resulting in accumulation of chitin to counteract cell wall abnormalities. Moreover, overexpression of *gfa1* resulted in a roughly threefold elevation in chitin levels [13]. Similarly, in *Aspergillus niger*, disruption of *gfaA* gene underscores its indispensable nature, as evidenced by growth being dependent on glucosamine supplementation. The upregulation of *gfaA* and the subsequent increase in chitin production represents ubiquitous compensatory strategies aimed at maintaining cell wall integrity during stress conditions. Additionally the study in *A. niger* underscores the pivotal involvement of *gfaA* in spore germination and viability [14].

In this study, we elucidate the critical role of *gfa1* in *A. fumigatus* by constructing a knockout mutant. We found that the Δ*gfa1* mutant depends on exogenous GlcN or GlcNAc for survival and exhibits abnormalities in growth, conidiation, and germination under partial repression (PR) conditions. The mutant displayed reduced chitin content under PR conditions, making it more vulnerable to cell wall, cell membrane, and endoplasmic reticulum stressors. Additionally, the mutant also exhibited disruptions in iron acquisition and metabolism, stemming from the inefficient conversion of L-glutamine (L-Gln) to L-glutamate (L-Glu), and exhibited attenuated virulence in both *Caenorhabditis elegans* and *Galleria mellonella* infection models. These findings highlight *gfa1* as a promising antifungal target, given its essential role in *A. fumigatus* physiology and pathogenesis.

## Results

### *gfa1 *is essential for *A. fumigatus* survival

Blastp searches using the *S. cerevisae* Gfa1 (Uniprot ID: P14742) identified a single Gfa1 protein in *A. fumigatus* (Uniprot ID: B0Y718). Phylogenetic analysis of the *A. fumigatus* Gfa1 protein indicates its closest relation to *A. niger* Gfa1, shared an identity of 96.40% (Additional file 1: Fig. S1A). The *gfa1* gene in *A. fumigatus* (AFUB_072250) spans a length of 2417 bp, with a coding sequence of 2085 bp. This sequence encodes a Gfa1 protein consisting of 694 amino acids, characterized by three conserved domains: two sugar isomerase (SIS) domains and one glutamine amidotransferase domain (Additional file 1: Fig. S1B). The identity of *A. fumigatus* Gfa1 with Gfa1 proteins from various species, including *S. cerevisae, Candida albicans, C. auris, Cryptococcus neoformans, Homo sapiens* and *Mus musculus,* ranges from 55 to 64% (Additional file 1: Fig. S1C).

**Fig. 1:**
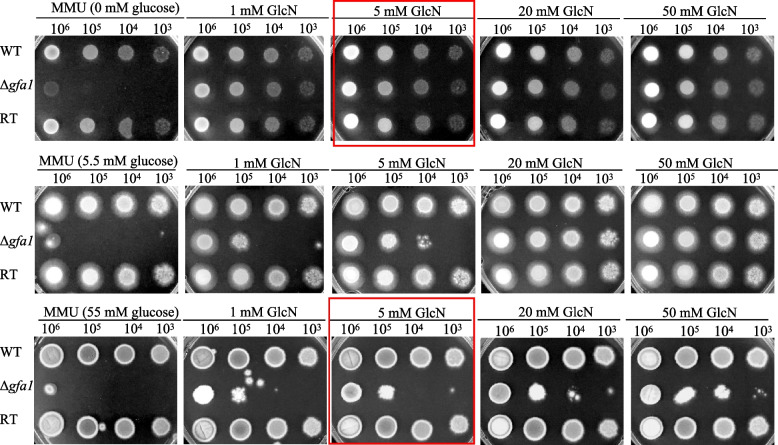
Growth of the Δ*gfa1* mutant at different concentrations of glucose and GlcN. Conidia at a concentration of 10^3^ to 10^6^ from the WT, Δ*gfa1*, and RT strains were grown on MMU with combinations of different carbon sources: (i) 0 mM Glc with 1 mM to 50 mM GlcN, (ii) 5.5 mM Glc with 1 mM to 50 mM GlcN, (iii) 55 mM Glc with 1 mM to 50 mM GlcN. Plates were incubated at 37 °C and photographed after 48 h. The 5 mM GlcN condition is referred to as the full recovery condition (FR), while the condition with 5 mM GlcN and 55 mM Glc condition is referred to as the partial repression condition (PR)

To investigate the function of *A. fumigatus gfa1*, we generated a knockout mutant strain through a homologous recombination strategy (Additional file 1: Fig. S2A). The knockout mutant was selected on regeneration plates with GlcN as the carbon source. Confirmation of the Δ*gfa1* and revertant (RT) strains was achieved through PCR using six primer pairs and Southern blotting (Additional file 1: Fig. S2B and Additional file 1: Fig. S2C). Similar to the study in *A. niger* [14], the *gfa1* mutant in *A. fumigatus* exhibited viability only in media supplemented with GlcN or GlcNAc. To further investigate the impact of glucose on growth of the mutant strain, we prepared media containing various concentrations of glucose in combination with GlcN or GlcNAc, and evaluated the growth patterns accordingly. Optimal growth of the mutant was observed when the medium contained 5 mM GlcN or 5 mM GlcNAc but no glucose (Fig. 1 and Additional file 1: Fig. S3). This suggests that *A. fumigatus* can utilize both GlcN and GlcNAc, with 5 mM GlcN being sufficient to counteract the effects of *gfa1* deficiency. However, as glucose concentration increased, the mutant exhibited a gradual reduction in growth compared to the no-glucose condition. We defined the medium containing 5 mM GlcN as the sole carbon source as the full recovery condition (FR), and the medium with 55 mM glucose and 5 mM GlcN as the partial repression condition (PR). These two conditions were selected for further functional analysis.

### *gfa1* deletion leads to reduced growth, decreased conidiation, and defective germination

**Fig. 2:**
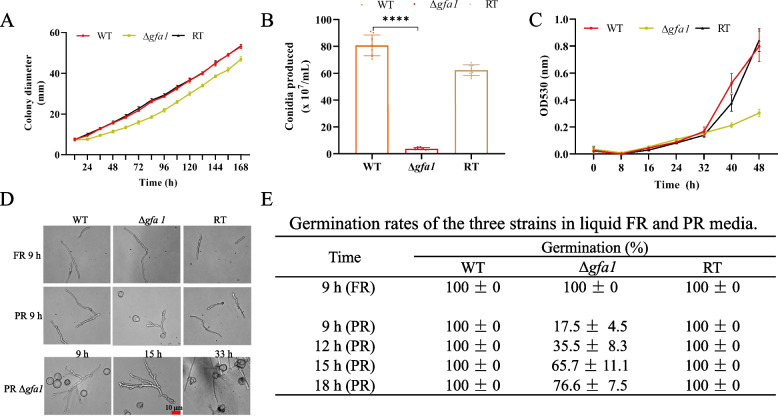
The growth phenotype of the Δ*gfa1* mutant under PR conditions. **A** Colony diameter measurements were taken at 12 h intervals over 7 days at 37 °C after inoculating conidia from the WT, Δ*gfa1*, and RT strains onto PR plates. The data represent the means ± SD from three independent replicates. **B** After 7 days, conidia were harvested and counted using a hemocytometer. Asterisk indicate a significant difference (multiple* t* test, ****, *p* < 0.0001). **C** The radial extension rate in liquid PR was continuously monitored using a plate reader for 48 h at 37 °C. Values represent means ± SD from three independent replicates.. **D** Germination was examined using a differential interference contrast microscope (Leica) at indicated time points during cultivation at 37 °C. **E** The germination rate of one hundred conidia of each strain was calculated, and values represent the mean ± SD

To elucidate the functional significance of *gfa1*, we conducted a phenotypic analysis of the mutant strain under PR condition and compared its phenotype to both WT and RT strains. As shown in Fig. 2, the Δ*gfa1* mutant exhibited reduced colony diameter, decreased radial extension rate, and low conidial production (Fig. 2A-C). Additionally, the mutant conidia exhibited aberrant morphology and a delayed germination rate (Fig. 2D). Under FR conditions, nearly all conidia from the three strains germinated after 9 h of incubation. However, under PR conditions, only 17.5% of mutant conidia had germinated by 9 h, with 23.4% remaining un-germinated even after 18 h, whereas 100% of WT and RT conidia had germinated by 9 h (Fig. 2E). Notably, the mutant conidia displayed abnormal polarity, characterized by multiple branches or swollen conidia without germ tubes (Fig. 2D).

### *gfa1* deletion affects cell wall integrity and stress resistance in *A. fumigatus*

**Fig. 3:**
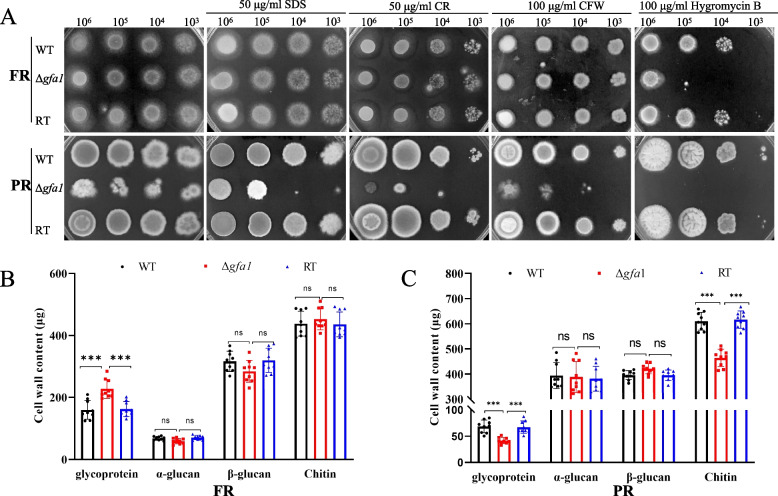
Cell wall integrity and component analysis of the Δ*gfa1* mutant. **A** Sensitivity of the Δ*gfa1* mutant to cell wall and cell membrane perturbing agents under FR and PR conditions. **B** Analysis of cell wall components in the three strains was performed under FR conditions. The values represent means ± SD from three replicates, and *p* values were calculated using multiple t tests (***, *p* < 0.001). **C** Analysis of cell wall components in the three strains was conducted under PR conditions. The values represent means ± SD from three replicates, and *p* values were calculated using multiple t tests (***, *p* < 0.001)

Chitin, constituting 10–20% of the dry weight of the fungal cell wall, provides critical mechanical strength. Given Gfa1's essential role in UDP-GlcNAc biosynthesis-an indispensable precursor for chitin production-we hypothesized that deleting *gfa1* would impair cell wall integrity and increase susceptibility to stress. To test this, we assessed the sensitivity of the Δ*gfa1* mutant to various stress-inducing agents, including cell wall perturbing agents (CR and CFW), a cell membrane disruptor (SDS), and a protein synthesis inhibitor (hygromycin B). Under PR conditions, Δ*gfa1* exhibited heightened sensitivity to cell wall and cell membrane stressors, as well as hygromycin B. In contrast, no significant sensitivity was observed under FR conditions, except for hygromycin B (Fig. 3A). Analysis of cell wall components revealed that, under FR conditions, the chitin content remained comparable between ∆*gfa1* and WT, while cell wall glycoprotein levels were elevated in the mutant (Fig. 3B). However, under PR conditions, the mutant exhibited significantly reduced chitin and glycoprotein levels compared to WT (Fig. 3C). These results suggest that deleting *gfa1* disrupts both cell wall and membrane integrity in *A. fumigatus*. Moreover, under PR conditions, Δ*gfa1* exhibited increased sensitivity to various antifungal drugs, including itraconazole, amphotericin B (AmB), fluconazole, and micafungin, as well as osmotic stabilizers and oxidative stress (Additional file 1: Fig. S4). These findings underscore the importance of *gfa1* in maintaining *A. fumigatus* survival and resistance under adverse conditions.

### *gfa1* deletion affects protein glycosylation and thermotolerance

**Fig. 4:**
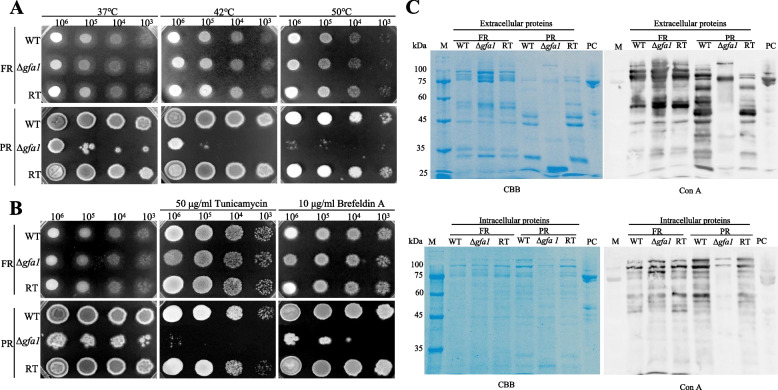
Role of *gfa1* in thermotolerance and protein glycosylation. **A** Sensitivity of the Δ*gfa1* mutant to high temperatures on solid PR or FR media incubated at 37 °C, 42 °C, or 50 °C for 48 h; **B** Sensitivity of the Δ*gfa1* mutant to ER stressors on solid PR or FR media supplemented with 50 μg/ml TM and 10 μg/ml brefeldin A, and incubated at 37 °C for 48 h. **C** Western blotting analysis of extracellular and intracellular proteins from the WT, Δ*gfa1*, and RT strains using a biotinylated ConA antibody, PC refers to the positive control, which is the Crf protein with N-glycosylation detectable by ConA. CBB staining served as the protein loading control

Glycosylation plays a crucial role in conferring thermotolerance. A substantial increase in protein O-GlcNAcylation has been reported in mammalian cells during acute heat stress, closely linked to enhanced cell survival under hyperthermic conditions [15]. Since Gfa1 is involved in the biosynthesis of UDP-GlcNAc, a key substrate for N-linked protein glycosylation [12], we investigated the impact of *gfa1* deletion on thermotolerance in *A. fumigatus*. As shown in Fig. 4A and B, under FR conditions, the WT, *∆gfa1,* and RT strains were thermally stable and exhibited similar sensitivity to the ER stressors tunicamycin (TM) and brefeldin A. However, the *∆gfa1* strain exhibited reduced growth at higher temperatures (42 °C and 50 °C) and increased sensitivity to TM and brefeldin A under PR conditions. To further investigate protein and glycoprotein changes, we performed Coomassie Brilliant Blue (CBB) staining and ConA antibody assays. The CBB assay showed no significant difference in total protein content between WT and *∆gfa1* under both FR and PR conditions. However, the ConA assay revealed a marked reduction in both secreted and intracellular glycoproteins in the *∆gfa1* under PR conditions, whereas no significant changes were observed under FR conditions (Fig. 4C). These findings indicate that the loss of *gfa1* compromises glycoprotein production, contributing to impaired thermotolerance and heightened sensitivity to ER stress under PR conditions.

### Transcriptomic analysis reveals regulatory dynamics of *gfa1* deletion in *A. fumigatus* physiology

**Fig. 5:**
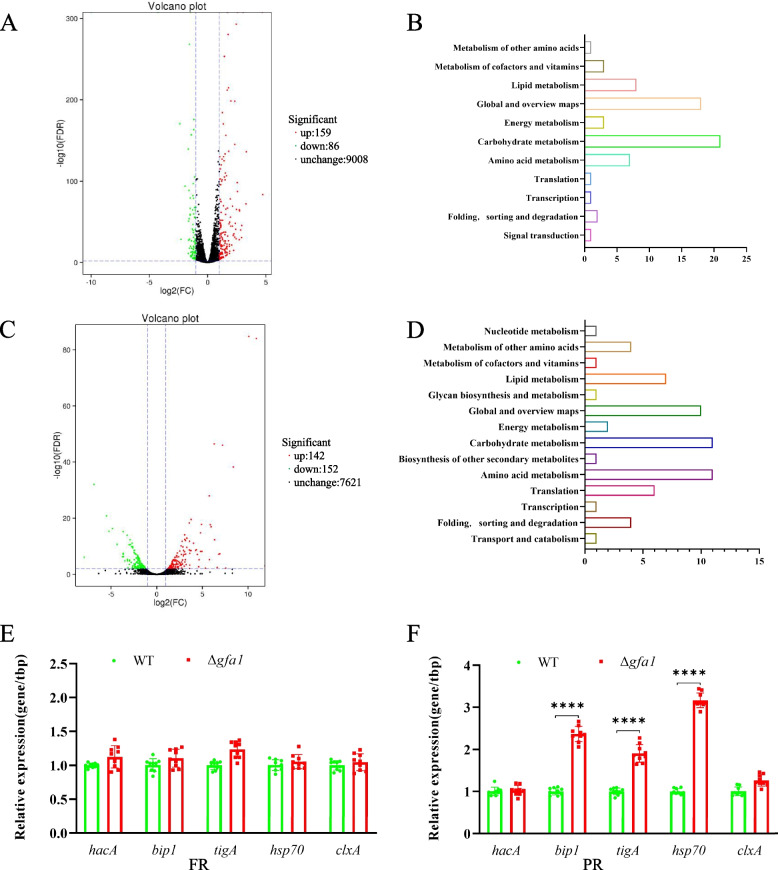
Transcriptomic analysis of the WT and Δ*gfa1* strains. **A** A volcano plot displaying the differential gene expression between WT and Δ*gfa1* strains under the FR condition. **B** KEGG category functional annotation showing the distribution of differentially expressed genes between the two strains under the FR condition. **C** A volcano plot displaying the differential gene expression between WT and Δ*gfa1* strains under the PR condition. **D** KEGG category functional annotation showing the distribution of differentially expressed genes between the two strains under the PR condition. **E** The qRT-PCR analysis of ER stress response genes (*hacA, bip1, tigA, hsp70,* and *clxA*) under FR and (**F**) PR conditions. Error bars represent the SD from three independent experiments with three replicates. (**** *p* < 0.0001)

To investigate the impact of *gfa1* deletion on gene expression, we performed transcriptomic analysis comparing the WT and ∆*gfa1* strains. Differentially expressed genes (DEGs) were identified using DESeq2 with a fold change ≥ 2 and FDR < 0.01. The ∆*gfa1* strain displayed significant changes in gene expression profiles under both FR and PR conditions. Specifically, 245 DEGs were identified under FR conditions, with 159 genes upregulated and 86 genes downregulated (Fig. 5A). Similarly, under PR conditions, 294 DEGs were identified, including 142 upregulated and 152 downregulated genes (Fig. 5C). KEGG pathway analysis revealed disruptions in carbohydrate metabolism under both conditions, with additional perturbations in amino acid metabolism under PR conditions (Fig. 5C and D). Notably, genes encoding chitinases and glucanases, key enzymes in cell wall remodeling and stress response[16], were downregulated in the ∆*gfa1* strain, likely contributing to its hypersensitivity to cell wall stressors (Additional file 2: Table S1). Moreover, we identified downregulation of genes involved in post-transcriptional modification, such as mannosidase Msds, amino peptidase, signal peptidase, stomatin family protein, and secretion pathway protein Sls2/Rcy1 (Additional file 2: Table S2), which may contribute to the reduced levels of extracellular and intracellular glycoproteins in the mutant under PR conditions. In response to cellular stress from misfolded and toxic proteins, cells activate the UPR pathway [17]. Our findings revealed relative upregulation of UPR-associated genes such as *bip1, tigA,* and *hsp70* in the *∆gfa1* mutant compared to WT under PR conditions (Fig. 5F), suggesting activation of the UPR to counteract proteotoxic stress. These findings highlight the intricate regulatory networks influenced by *gfa1* deletion, elucidating its multifaceted role in cell wall integrity, protein homeostasis, and stress response in *A. fumigatus*.

### *gfa1* is involved in iron acquisition and metabolism in *A. fumigatus*

**Fig. 6:**
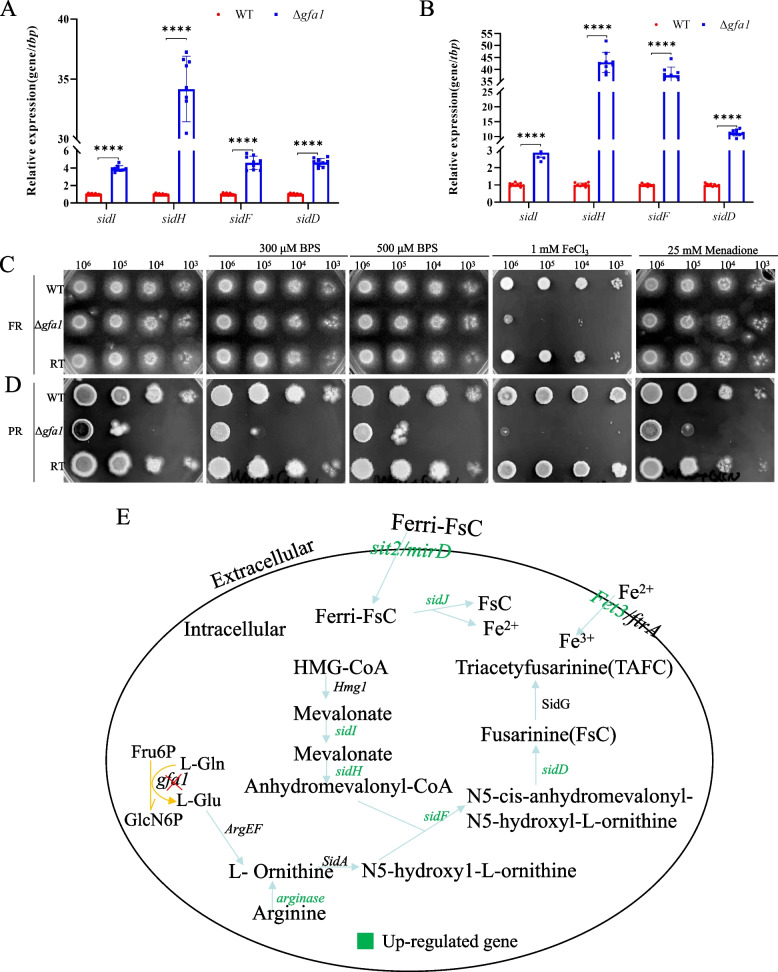
*gfa1* is important for iron acquisition and metabolism. **A** qRT-PCR analysis the expression levels of *sidI, sidH, sidF* and *sidD* in FR media. Error bars represent the SD from three independent experiments with three replicates (**** *p* < 0.0001). **B** The expression levels of *sidI, sidH, sidF,and sidD* in PR media by qRT-PCR assay. Error bars represent the SD from three independent experiments with three replicates (**** *p* < 0.0001). **C** Freshly harvested conidia (10^6^ to 10^3^) were cultured on FR medium supplemented with 300 μM BPS, 500 μM BPS, 1 mM FeCl_3_, and 25 mM Menadione. Plates were incubated at 37 °C for 48 h. **D** Serially diluted conidia were cultured on PR medium supplemented with 300 μM BPS, 500 μM BPS, 1 mM FeCl_3_, and 25 mM Menadione and incubated at 37 °C for 48 h. **E** Schematic representation of the iron acquisition and metabolism in Δ*gfa1* mutants under PR condition and FR condition

Transcriptomic analysis revealed upregulation of key siderophore biosynthesis genes, including *sidI, sidH, sidF, and sidD,* in the Δ*gfa1* mutant under both FR and PR conditions (Additional file 2: Table S3)*.* Notably, the expression of transcription factors *hapX* and *sreA*, which are critical regulators of fungal iron homeostasis, remained unaltered in both WT and Δ*gfa1* strains under these conditions (Additional file 2: Table S3). These findings were further supported by qRT-PCR, which consistently demonstrated upregulation of siderophore biosynthesis genes (Fig. 6A and B).

Iron is indispensable for the survival and pathogenicity of *A. fumigatus*, with its uptake primarily mediated by siderophores [18, 19]. Gfa1 catalyzes the conversion of glutamine to glutamate, a critical step in siderophore biosynthesis [20]. Functional assays showed that the Δ*gfa1* mutant was unable to grow in excess iron (FeCl_3_), suggesting defective iron metabolism and a susceptibility to iron toxicity under both FR and PR conditions. However, the mutant exhibited no significant hypersensitivity to the iron-chelating agent BPS or the oxidative stressor menadione under these conditions (Fig. 6C and D). Furthermore, in addition to the upregulation of *sidI*, *sidH*, *sidF*, and *sidD*, the Δ*gfa1* mutant also exhibited elevated expression of genes encoding siderophore-iron transporters (*sit2*, *mirD)*, *arginase* and components of reductive iron assimilation (*Fet3*), as revealed by our transcriptomic data (Fig. 6E and Additional file 2: Table S3). These findings suggest that the mutant cells perceived a state of iron deficiency and responded by upregulating iron acquisition pathways, starting with the activation of arginine to ornithine. However, this apparent iron deficiency is likely attributable to the depletion of glutamate caused by the loss of *gfa1* activity. In summary, *gfa1* plays a pivotal role in regulating iron acquisition and metabolism in *A. fumigatus*, highlighting its importance in maintaining iron homeostasis and overall fungal fitness.

### *gfa1* deletion results in attenuated virulence

**Fig. 7:**
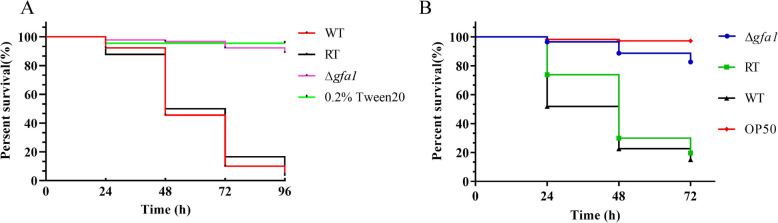
Virulence tests of *A. fumigatus* strains in *G. mellonella* and *C. elegans* models. **A** Kaplan–Meier survival plot of *G. mellonella* larvae at 24, 48, 72, and 96 h after injection with conidia of the indicated strains and incubation at 37 °C. For each strain, three biological repeats (each with triplicates) were conducted. **B** Kaplan–Meier survival plot of glp-4 (bn2) and sek-1 (km4) worms fed by the conidia of the indicated strains after 16 h of infection. Worms infected with the *Escherichia coli* OP 50 strain were used as controls. For each strain, three biological repeats (each in triplicate) were conducted

As the primary causative agent of aspergillosis, *A. fumigatus* relies on various critical processes, including cell wall biogenesis, protein homeostasis, oxidative stress response, and iron metabolism, for virulence. Given the essential role of *gfa1* in these pathways, we assessed its contribution to *A. fumigatus* virulence using *G. mellonella* and *C. elegans* infection models. Survival rates of worms infected with WT, Δ*gfa1*, and RT strains were monitored and analyzed using Kaplan–Meier survival curves. The Δ*gfa1* mutant exhibited significantly reduced virulence compared to the WT and RT strains as shown in Fig. 7A. In *C. elegans*, the Δ*gfa1* mutant caused markedly lower mortality, mirroring similar attenuated virulence trends observed in *G. mellonella* (Fig. 7B). These findings highlight the crucial role of *gfa1* in *A. fumigatus* pathogenicity, underscoring its importance as a potential target for antifungal therapies.

## Discussion

Each year, invasive fungal diseases affect over 6.5 million individuals globally, resulting in approximately 3.8 million deaths [2]. Currently, only four main classes of medications-polyenes, 5-fluorocytosine, echinocandins, and azoles-are approved for human therapy [5]. The growing menace of drug-resistant infections presents a substantial and expanding societal and economic challenge. Therefore, there is an urgent need for innovative strategies to prevent fungal infections [21].

The cell wall is vital for maintaining cellular stability, regulating permeability, and providing stress resistance, emphasizing the need to understand its composition and biogenesis [22]. The chemicals that compose the fungal cell wall are largely absent in the human body, making them excellent targets for developing immunotherapies and antifungal drugs [8]. Chitin, a linear polymer of GlcNAc units linked by β-(1–4) glycosidic bonds, typically constitutes 10–20% of the cell wall in filamentous fungi [8]. The HBP converts Fru6P and glutamine into glucosamine-6-phosphate (GlcN6P) and glutamate, playing a crucial role in generating UDP-GlcNAc through four enzymatic steps. UDP-GlcNAc is an important amino sugar donor for protein and lipid glycosylation and GPI-anchor, as well as for chitin production [23]. Enzymes involved in the HBP, such as GNA1, AGM1, and UAP1, have been identified as promising targets for antifungal drugs [24–26].

In this study, we elucidate the critical role of Gfa1 in *A. fumigatus*, which catalyzes the rate-limiting step in the UDP-GlcNAc pathway. Previous studies have shown that supplementing the medium with exogenous GlcN can rescue the *gfa1* deletion mutant in *A. niger* [14]. Similarly, our findings corroborate that *A. fumigatus* cannot survive without *gfa1* unless the medium is supplemented with exogenous GlcNAc or GlcN (Fig. 1 and Additional file 1: Fig. S3). The Δ*gfa1* mutant's growth cannot be sustained by glucose alone; however, providing GlcN or GlcNAc as the sole carbon sources supports optimal growth. When both glucose and GlcN/GlcNAc are present in the medium, the mutant exhibits variable growth patterns. Specifically, higher glucose concentrations correlate with poorer growth of the mutant, suggesting a suppressive effect on GlcN/GlcNAc uptake by glucose. This suppression is likely due to carbon catabolite repression [27] and competition for sugar transporter affinity, which requires further investigation.

The role of *gfa1* in increased chitin deposition in the cell walls has been previously reported in other fungi [14]. Given the crucial role of chitin in cell wall stability [28], we investigated the cell wall integrity of the Δ*gfa1* mutant under cell wall stressors CR and CFW. Unlike the FR conditions, the Δ*gfa1* mutant exhibited hypersensitivity to cell wall stressors under PR conditions (Fig. 3A). Analysis of cell wall components revealed significantly lower chitin contents in the mutant compared to WT and RT strains (Fig. 3C), demonstrating the *gfa1* deficiency leads to defect cell wall due to insufficient chitin levels. A higher flux through the HBP pathway ensures the production of sufficient UDP-GlcNAc for proper chitin level [13]. It can be conclude that adequate GlcN intake under FR conditions ensures proper chitin levels in the Δ*gfa1* mutant (Fig. 3B), whereas reduced chitin levels occur under PR conditions due to insufficient GlcN for the HBP pathway, caused by glucose suppression. Moreover, our transcriptomic data revealed the downregulation of chitinases and glucanases (Additional file 2: Table S1), which are associated with stress responses [16, 29], indicating a potential decrease in the mutant’s ability to remodel its cell wall, rendering it more sensitive to external perturbations and stressors.

Protein glycosylation is essential for proper protein folding, stability, and functionality, particularly through N-linked glycosylation in the ER and subsequent N-glycan remodeling in the Golgi. UDP-GlcNAc serves as the donor for protein glycosylation, with Gfa1 acting as the rate-limiting enzyme in its biosynthesis. Gain-of-function mutations (G451E) in *Caenorhabditis elegans* GFAT-1 elevate UDP-GlcNAc levels, enhancing protein homeostasis, lifespan, and tunicamycin resistance [30]. Similarly, in *Arabidopsis*, overexpression of *At*GFAT1 increases glucosamine and tunicamycin resistance, while RNAi suppression has the opposite effect [31]. In our study, under PR conditions, insufficient GlcN reduced HBP flux and UDP-GlcNAc production, inducing ER stress and the UPR. This was evidenced by increased sensitivity to ER stressors tunicamycin and brefeldin A (Fig. 4B), upregulation of UPR-associated genes *bip1*, *tigA*, and *hsp70* (Fig. 5F), and downregulation of genes involved in post-transcriptional modification, such as mannosidase Msds, aminopeptidase, signal peptidase, stomatin family protein, and secretion pathway protein Sls2/Rcy1 (Additional file 2: Table S2). ConA assays revealed reduced glycoprotein levels in the mutant under PR conditions, which likely contributed to increased sensitivity to high temperatures (42 °C and 50 °C), underscoring the importance of glycosylation for thermotolerance and viability in *A. fumigatus* [15].

UDP-GlcNAc also serves as a precursor for glycosylphosphatidylinositol (GPI) anchor synthesis [32], a conserved post-translational modification in eukaryotes that attaches proteins to the plasma membrane and cell wall [33]. In yeast, many GPI-anchored proteins are essential for morphogenesis and cell wall organization. Similarly, in *A. fumigatus*, disruption of GPI anchor synthesis results in severe phenotypes, including cell wall defects, increased cell death, and attenuated virulence [34]. In our study, under FR conditions, the availability of sufficient GlcN enhances HBP flux, leading to increased UDP-GlcNAc production. This, in turn, results in elevated cell wall glycoprotein levels in the Δ*gfa1* mutant compared to the WT strain (Fig. 3B). Conversely, under PR conditions, limited GlcN reduces HBP flux and UDP-GlcNAc production, compromising cell wall integrity and leading to a significant decrease in cell wall glycoprotein levels in the Δ*gfa1* mutant compared to the WT strain (Fig. 3A and C). These findings highlight the critical role of UDP-GlcNAc in maintaining cell wall integrity and glycoprotein levels in *A. fumigatus*.

Fungi must regulate iron acquisition, storage, and utilization to maintain iron homeostasis, crucial for survival and virulence in host environments where iron is limited [19]. In *A. fumigatus*, two high-affinity iron acquisition mechanisms-reductive iron assimilation (RIA) and siderophore-mediated uptake-are upregulated in response to iron deficiency [19]. Ornithine, a non-proteinogenic amino acid, is a key precursor for siderophores, produced either in the mitochondria as an intermediate of arginine biosynthesis and exported to the cytosol by AmcA, or directly in the cytosol via arginase-mediated arginine hydrolysis. Our transcriptome data and qRT-PCR results showed upregulation of siderophore synthesis genes (*sidI*, *sidH*, *sidF*, and *sidD*) under both FR and PR conditions (Fig. 6A and B). Extracellular siderophores capture Fe^3+^ and transport it across the fungal membrane via siderophore-iron transporters (SITs). Our data also indicated upregulation of genes encoding these transporters (*sit2* and *mirD*) (Fig. 6E and Additional file 2: Table S3). Additionally, the RIA gene *Fet3* was upregulated, suggesting activation of both RIA and siderophore-mediated uptake in the Δ*gfa1* mutant. The Δ*gfa1* mutant's hypersensitivity to 1 mM FeCl_3_ (Fig. 6C and D), compared to the WT, implies enhanced iron uptake, leading to iron toxicity under both FR and PR conditions. Transcriptional regulators *SreA* and *HapX*, as well as *SrbA*, *AcuM*, and *AcuK*, are crucial for iron homeostasis. However, our data showed no altered expression of these regulators in the Δ*gfa1* mutant compared to WT strains under FR and PR conditions (Additional file 2: Table S3), indicating that Gfa1 regulates iron metabolism independently of these transcription factors. Notably, this study is the first to identify a gene involved in central carbon metabolism as a negative regulator of iron ion metabolism, shedding new light on the interplay between metabolic pathways and iron homeostasis.

Gfa1 is the primary rate-limiting enzyme in UDP-GlcNAc synthesis, controlling HBP flux, and is commonly targeted by glutamine analog inhibitors [35–37]. However, the virulence of Gfa1 mutants in hosts is rarely documented. Translating in vitro phenotypic characterizations into in vivo infection models is crucial for validating genetic targets for antifungal therapies. It is important to determine if ubiquitous amino sugars can compensate for the loss of Gfa1 in vivo. For instance, *Leishmania major* Δ*gfat* promastigotes cannot proliferate in vitro infected macrophages, while Δ*gfat* amastigotes grow like wild-type amastigotes in macrophages and cause lesions in susceptible mice, suggesting they can scavenge glucosamine from the macrophage phagolysosome [38]. In our study, Δ*gfa1* mutants exhibited reduced virulence in *G. mellonella* and *C. elegans* infection models, although their phenotype was rescued by amino sugars in vitro. This is the first report indicating that Gfa1 contributes to *A. fumigatus* pathogenesis in animal models. The compelling evidence suggests that Gfa1 has substantial potential as a promising drug target.

## Conclusions

**Fig. 8:**
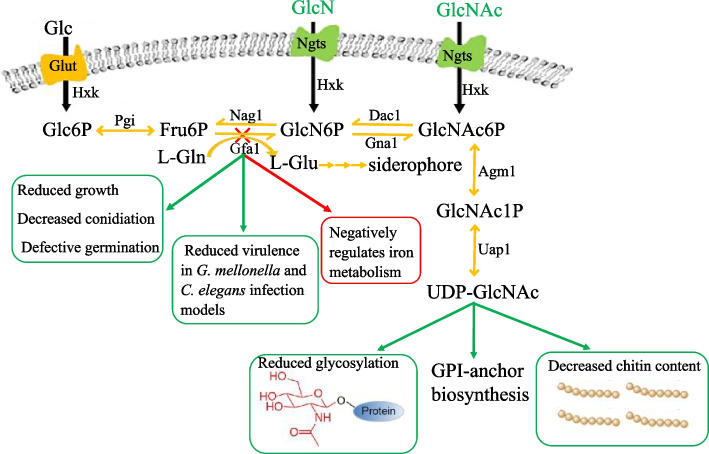
The schematic diagram demonstrates the pleiotropic effects of *gfa1* in *A. fumigatus*, highlighting its impact on chitin biosynthesis, cell wall integrity, protein glycosylation, and iron metabolism

In summary, knockout of *gfa1* significantly impacted multiple cellular processes in *A. fumigatus* under PR conditions, including chitin biosynthesis, cell wall integrity, protein glycosylation, and iron metabolism (Fig. 8). Although supplementation with amino sugars effectively restored the Δ*gfa1* phenotype in vitro, the pathogenicity of the Δ*gfa1* mutant was significantly reduced in both *G. mellonella* and *C. elegans* infection models. Our study comprehensively elucidates the pleiotropic effects of *gfa1* in *A. fumigatus*, providing compelling evidence that *gfa1* represents a promising drug target against this pathogen.

## Methods

### Strains and culture conditions

The *A. fumigatus* Δ*ku80pyrG-* strain served as the parental strain for transformation, while Δ*ku80* represented the wild type (WT) for phenotypic analysis. Cultures of these strains were maintained at 37 °C on complete medium (CM) or minimal medium (MM) supplemented with 5 mM uridine and uracil (MMU) if required. The *gfa1* mutant strain was sustained on MM medium supplemented with 55 mM glucose and 5 mM GlcN as the partial repression (PR) condition for functional analysis. Mycelia were harvested from liquid medium at 37 °C with 200 rpm shaking at specified time points, washed with distilled water and then rapidly frozen in liquid nitrogen. The mycelia powder was stored at −80 °C for DNA and RNA extraction. Spores were collected from two-day-old cultures incubated at 37 °C using 0.2% (v/v) Tween 20 in water.

### Construction of the *A. fumigatusgfa1* mutant and revertant strains

The *gfa1* deletion mutant was generated through a homologous recombination strategy [39]. Upstream and downstream flanking sequences of approximately 1 kb each from the *gfa1* gene were first amplified from the WT strain using primer pairs P1/P2 and P3/P4, respectively. Subsequently, the upstream fragment was digested with *Asc*I/*Not*I and the downstream fragment was digested with *Fse*I/*Pac*I. Meanwhile, the neo-*pyrG-neo* selection marker [40] was amplified from the pCDA14 plasmid and digested with *Not*I and *Fse*I. The three fragments were then ligated together with the appropriately digested pBlueScript II-modified SK plasmid to generate the *gfa1* knockout vector, which was subsequently verified through sequencing. The linearized *gfa1* knockout fragment was used to transform Δ*ku80pyrG-* protoplasts via polyethylene glycol (PEG)-mediated fusion [41]. Positive transformants were selected on MM supplemented with GlcN as the sole carbon source. Confirmation of the transformants was carried out through PCR using six different primer pairs (P5/P6, P7/P8, P9/P2, P3/P10, P9/P11, and P12/P10). All the primers used in this study are listed in Additional file 2: Table S1.

For complementation of the *gfa1* null mutant, a full-length *gfa1* gene was amplified using the primer pair P1/P4. The recombinant plasmid c-*gfa1* was constructed by the insertion of the *gfa1* gene to replace the neo-*pyrG-neo* fragment of the *gfa1* vector, and the resulting vector was transformed into the Δ*gfa1* protoplast. Transformants were screened on MMU medium and confirmed by PCR, following the same procedure as for confirming the Δ*gfa1* mutant.

The *∆gfa1* and revertant (RT) strains were also confirmed by Southern blot analysis, using upstream fragment and *pyrG* marker as probes. For this analysis, 20 μg of genomic DNA from the WT, Δ*gfa1,* and RT strains was digested with *KpnI* and *XhoI* for the upstream and *pyrG* probes, respectively. The labelling and visualization processes were conducted employing the digoxigenin (DIG) DNA labelling and detection kit (Roche Applied Science), following the manufacturer’s guidelines.

### Growth phenotype, conidia production, and germination morphology analysis of the *gfa1* mutant

To investigate the impact of glucose and GlcN/GlcNAc on growth, freshly harvested serially diluted conidia (10^6^ to 10^3^) of the WT, Δ*gfa1,* and RT strains were individually point inoculated on MMU plates containing varying concentrations of glucose (0, 5.5, and 55 mM) and GlcN/GlcNAc (1, 5, 20, and 50 mM). The plates were incubated at 37 °C and photographed after 48 h.

For assessment of radial extension rates, conidia from all three strains at a concentration of 2 × 10^4^ were incubated in 200 µl of FR or PR medium in 96-well plates and incubated at 37 °C. The OD_530_ was measured every 8 h over a continuous 48 h period using a Tecan i-control microplate reader. The experiment was conducted with three biological replicates and three technical replicates each.

Conidiation analysis involved point inoculating conidia of all three strains at a concentration of 1 × 10^6^ into the center of plates containing FR or PR medium. Colony diameter was measured twice daily along the same marked line until the entire plate was covered by the colony. Spores were harvested individually from all three strains and counted using a hemocytometer.

For the conidia germination morphology assay, 2 × 10^5^ conidia of the WT, Δ*gfa1,* and RT strains were incubated on glass coverslips in a 6-well plate with 4 ml of PR and FR at 37 °C for specified durations (9, 15, and 33 h). Images were captured using an inverted microscope (Leica).

### Stress sensitivity assays

For sensitivity assays encompassing cell wall, cell membrane, oxidative, and osmotic stressors, along with antifungal drugs, freshly harvested serially diluted conidia (10^6^–10^3^) of the WT, Δ*gfa1,* and RT strains were spotted onto FR and PR medium plates. These plates contained specified concentrations of stressors and antifungal drugs, including calcofluor white (CFW), congo red (CR), sodium dodecyl sulfate (SDS), hygromycin B, NaCl, sorbitol, H_2_O_2_, itraconazole, amphotecerin B (AmB), fluconazole, and micafungin. The plates were incubated at 37 °C and photographed after 48 h.

For temperature sensitivity assessment, serially diluted conidia (10^6^–10^3^) of the WT, Δ*gfa1,* and RT strains were spotted onto FR and PR plates and subjected to incubation at 37 °C, 42 °C, 50 °C for 48 h, before photographic documentation. To evaluate sensitivity towards ER stress, (10^6^–10^3^) conidia of the WT, Δ*gfa1*, and RT strains were spotted onto FR and PR plates amended with ER stressors, tunicamycin (TM) and brefeldin A. The plates were incubated at 37 °C for 48 h before being photographed.

### Cell wall components analysis

Conidia of the WT, Δ*gfa1,* and RT strains were inoculated into PR and FR media and then incubated at 37 °C with shaking at 200 rpm for 48 h. The mycelia were harvested, washed thoroughly with water, and then subjected to freeze-drying. Cell wall extraction, polysaccharide hydrolysis, and quantification were conducted as described previously [42, 43]. Briefly, 10 mg of dried mycelium sample from each strain was put into 1 ml of 50 mM NH_4_HCO_3_ (pH 8.0) buffer and 0.2 g of stainless steel beads (1 mm) and the mixture was subjected to bead beating. After centrifugation, the resulting supernatant was discarded, and the pellet was washed four times with ddH_2_O. Next, the cell wall pellet was treated with 1 M KOH for 1 h at 70 °C and centrifuged to separate the alkali-soluble supernatant from the insoluble components. The supernatant containing α-glucans was collected by acidification with acetic acid, followed by centrifugation. The glycoproteins in the supernatant underwent protein concentration detection, ethanol precipitation, and subsequent digestion in 6 N HCl at 100 °C for 2 h to release monosaccharides. The alkali-insoluble (AI) components, comprising β-glucan and chitin, were also subjected to HCl hydrolysis [44–46]. The released glucose was quantified using the phenol–sulfuric acid method [47], while glucosamine was measured according to the method described by Lee et al. [48]. Three independent samples of lyophilized mycelial pads were used for cell wall analysis, and the experiment was repeated three times.

### Western blot analysis

For the total protein secretion analysis, conidia were inoculated into PR and FR media and incubated at 37 °C at 200 rpm for 3 days. The mycelia were harvested, ground, and suspended in an ice-cold extraction buffer (50 mM HEPES pH 7.4, 137 mM KCl, 10% glycerol, 1 mM EDTA, 1 μg/ml pepstatin A, 1 μg/ml leupeptin, and 1 mM PMSF). Following electrophoresis on a 12% SDS–PAGE gel, proteins were probed with biotinylated concanavalin A (Con A) as the primary antibody (Vector Labs) at a 1:2000 dilution, and streptavidin/FITC conjugates (Solarbio) served as the secondary antibody at a 1:400 dilution. Blots were acquired with the GE Typhoon FLA 9600 scanner.

### RNA isolation and quantitative real-time PCR

Fresh mycelia were harvested, ground into a fine powder. Total RNA was isolated using the Transzol up plus RNA Kit (Transgen, Beijing, China) according to the manufacturer’s instructions. For the qRT-PCR reactions, 2.5 μg of total RNA was reverse-transcribed using StarScript II First-strand cDNA kit (Gen Star, Beijing, China). Each cDNA sample was used for qPCR using the specific primer pairs listed in Table S1. qRT-PCR, was performed using ChamQ Universal SYBR qPCR Master Mix (Vazyme, Nanjing, Jiangsu, China) on an ABI 7500 System. The thermal cycling conditions were 40 cycles at 95 °C for 10 s for denaturation and 60 °C for 30 s for annealing and extension. Real-time PCR data were acquired using sequence detection software. The relative expression of each gene was calculated using the 2^−∆∆CT^ method [49]. Samples isolated from different strains at different times were tested in triplicate.

### Comparative transcriptomic analysis

Conidia from both the WT and Δ*gfa1* strains were inoculated into either FR or PR media and then incubated at 37 °C with shaking at 200 rpm for 48 h. Subsequently, the mycelia were harvested, washed thoroughly with ddH_2_O, and promptly frozen in liquid nitrogen before storage at −80 °C. RNA isolation and sequencing were conducted by Biomarker technologies (China). High-quality RNA samples were prepared, and sequencing was performed on the Illumina sequencing platform. Differentially expressed genes (DEGs) were identified using DESeq2 with a Fold Change ≥ 2 and FDR < 0.01. The visualization of DEGs was accomplished through volcano plots, whereas KEGG analysis was performed to characterize the biological functions of the DEGs and the metabolic pathways associated with these genes. The RNA-Seq Illumina reads were deposited in the National Center for Biotechnology Information Short Read Archive (NCBI-SRA) and are publicly available under the accession number PRJNA1129902.

### Virulence assessment

We conducted a comprehensive evaluation of the pathogenicity of the WT, Δ*gfa1*, and RT strains using a well-established virulence assay based on the *C. elegans* infection model [50]. Briefly, 10^8^ conidia from all the three strains were used to feed the worms at 25 °C. The pre-infected worms were put on the killing assay medium (brain heart infusion [BHI^+^]) at 20 °C after 16 h. Subsequently, survival rates were recorded at specified time (24, 48, and 72 h). The number of hyphal filaments that emerged from worm bodies were quantified by the hyphal filamentation rate. Worms fed with the *Escherichia coli* OP 50 strain were used as control.

For the *G. mellonella* virulence test [51], six instar larvae were chosen and divided into four groups, each containing 90 larvae. The hind pro-leg of each larvae was injected with 5 × 10^7^conidia from the WT, Δ*gfa1*, and RT strains using a Hamilton syringe. Larvae treated with 0.2% Tween-20 served as the control group. The survival rates of each group were monitored at 24, 48, 72 and 96 h post-infection. Dead larvae were those that showed signs of melanization or dark patches and were immobile.

## Supplementary Information

Additional file 1. Fig. S1. Phylogenetic analysis of Gfa1 proteins among different organisms. Gfa1 protein sequences from *Mus musculus*, *Rattus norvegicus*, *Homo sapiens*, *Danio rerio*, *Caenorhabditis elegans*, *Drosophilla* *melanogaster*,*Candida albicans*, *Saccharomyces cerevisae*, *Aspergillus flavus*,*Aspergillus fumigatus*, *Aspergillus niger*, *Arabidopsis thaliana*,*Vigna radiaae*, *Pseudomonos aeruginodsa*, *Esherica coli*, *Salmonella typhimorium*, *Bifdobacterium longum*, *Bacillus subtilis*, and *Helicobacter pylori* were downloaded from NCBI and analyzed using MEGA 11 neighbor joining method with a bootstrap value of 1,000 replicates.Domain prediction of the Gfa1 proteins by SMART. All Gfa1s have two SIS domains.The comparison of identities between *A. fumigatus* Gfa1 and Gfa1 proteins from other species. Fig. S2. A schematic representation of strategies for the construction and confirmation of the ∆*gfa1* and RT strains. A, Strategies for generating the Δ*gfa1* mutant by homologous recombination. B, PCR confirmation using six pairs of primers indicated in A. C, Verification of the Δ*gfa1 *mutant and RT strain by Southern blotting. WT is wild-type, Δ*gfa1* is the mutant, and RT is the revertant strain. Fig. S3. Growth of the Δ*gfa1* mutant at different concentrations of glucose and GlcNAc. Conidia at a concentration of 10^3^to 10^6 ^from the WT, Δ*gfa1*, and RT strains were grown on MMU with combinations of different carbon sources: 0 mM Glc with 1 mM to 50 mM GlcNAc, 5.5 mM Glc with 1 mM to 50 mM GlcNAc, 55 mM Glc with 1 mM to 50 mM GlcNAc. Plates were incubated at 37 °C for 48 hours. Fig. S4. Response of the Δ*gfa1* mutant to osmotic, oxidative stresses, and antifungal drugs. Conidia at 10^3^ to 10^6^ of the WT, Δ*gfa1*, and RT strains were cultured on FR or PR media supplemented with 0.8 M NaCl, 1.2 M sorbitol, 2 mM H_2_O_2_, 2 μg/ml itraconazole, 1 μg/ml AmB, 256 μg/ml fluconazole, and 2 μg/ml micafungin. Plates were maintained at 37 °C for 2 days. Fig. S5. Response of the Δ*gfa1* mutant to osmotic, oxidative stresses, and antifungal drugs. Conidia of the WT, Δ*gfa1*, and RT strains were cultured on media supplemented with 1 mM GlcN and 55 mM Glc or 1 mM GlcN, and incubated at 37 °C, 42 °C, or 50 °C for 48 h, or supplemented with 50 μg/ml SDS, 50 μg/ml CR, 100 μg/ml CFW, 100 μg/ml Hygromycin B, 1 mM FeCl_3_, and incubated at 37 °C for 48 h.

Additional file 2. Table S1.Changes in gene expression levels involved in cell wall biogenesis under PR conditions. Table S2 Changes in gene expression levels involved in protein posttranslational modification. Table S3 Changes in gene expression levels involved in iron metabolism. Table S4 Primers used in this study

## Data Availability

The authors confirm that all relevant data are included in the main manuscript and Additional files provided with the manuscript. The fASTO files associated with this proiect have been deposited in the NCBl database under accession number PRJNA1129902.

## Abbreviations

Gfa1: Glutamine fructose-6-phosphate aminotransferase
HBP: Hexosamine biosynthetic pathway
GlcN: Glucosamine
GlcNAc: N-acetylglucosamine
FR: Full recovery
WT: Wild-type
PR: Partial repression
ER: Endoplasmic reticulum
UPR: Unfolded protein response
IA: Invasive aspergillosis
CAPA: COVID-19-associated invasive pulmonary aspergillosis
WHO: World Health Organization
FPPL: Fungal priority pathogens list
Fru6P: Fructose-6-phosphate
L-Gln: L-glutamine
L-Glu: L-glutamate
SIS: Sugar isomerase
RT: Revertant
AmB: Amphotericin B
TM: Tunicamycin
CBB: Coomassie Brilliant Blue
DEGs: Differentially expressed genes
GlcN6P: Glucosamine-6-phosphate
GPI: Glycosylphosphatidylinositol
RIA: Reductive iron assimilation
CM: Complete medium
MM: Minimal medium
MMU: MM supplemented with 5 mM uridine and uracil
PEG: Polyethylene glycol
DIG: Digoxigenin
CFW: Calcofluor white
CR: Congo red
SDS: Sodium dodecyl sulfate
AI: Alkali-insoluble
Con A: Concanavalin A
NCBI-SRA: National Center for Biotechnology Information Short Read Archive
BHI^+^: Brain heart infusion
