# Supplementary material for: Gfa1 (glutamine fructose-6-phosphate aminotransferase) is essential for Aspergillus fumigatus growth and virulence
Source: BMC Biol. 2025 Mar 13;23:80. doi: 10.1186/s12915-025-02184-0 (PMC11907850; doi:10.1186/s12915-025-02184-0)
Supplement: Supplementary file 1 — Additional file 1. Fig. S1. Phylogenetic analysis of Gfa1 proteins among different organisms. Gfa1 protein sequences from Mus musculus, Rattus norvegicus, Homo sapiens, Danio rerio, Caenorhabditis elegans, Drosophilla melanogaster,Candida albicans, Saccharomyces cerevisae, Aspergillus flavus,Aspergillus fumigatus, Aspergillus niger, Arabidopsis thaliana,Vigna radiaae, Pseudomonos aeruginodsa, Esherica coli, Salmonella typhimorium, Bifdobacterium longum, Bacillus subtilis, and Helicobacter pylori were downloaded from NCBI and analyzed using MEGA 11 neighbor joining method with a bootstrap value of 1,000 replicates.Domain prediction of the Gfa1 proteins by SMART. All Gfa1s have two SIS domains.The comparison of identities between A. fumigatus Gfa1 and Gfa1 proteins from other species. Fig. S2. A schematic representation of strategies for the construction and confirmation of the ∆gfa1 and RT strains. A, Strategies for generating the Δgfa1 mutant by homologous recombination. B, PCR confirmation using six pairs of primers indicated in A. C, Verification of the Δgfa1 mutant and RT strain by Southern blotting. WT is wild-type, Δgfa1 is the mutant, and RT is the revertant strain. Fig. S3. Growth of the Δgfa1 mutant at different concentrations of glucose and GlcNAc. Conidia at a concentration of 103to 106 from the WT, Δgfa1, and RT strains were grown on MMU with combinations of different carbon sources: 0 mM Glc with 1 mM to 50 mM GlcNAc, 5.5 mM Glc with 1 mM to 50 mM GlcNAc, 55 mM Glc with 1 mM to 50 mM GlcNAc. Plates were incubated at 37 °C for 48 hours. Fig. S4. Response of the Δgfa1 mutant to osmotic, oxidative stresses, and antifungal drugs. Conidia at 103 to 106 of the WT, Δgfa1, and RT strains were cultured on FR or PR media supplemented with 0.8 M NaCl, 1.2 M sorbitol, 2 mM H2O2, 2 μg/ml itraconazole, 1 μg/ml AmB, 256 μg/ml fluconazole, and 2 μg/ml micafungin. Plates were maintained at 37 °C for 2 days. Fig. S5. Response of the Δgfa1 mutant to osmotic, oxidative stre [file 12915_2025_2184_MOESM1_ESM.pdf]

A

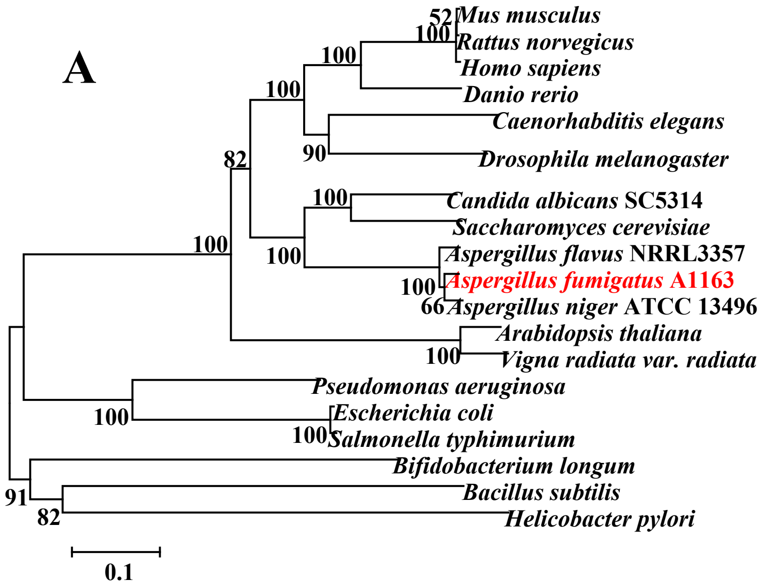

B

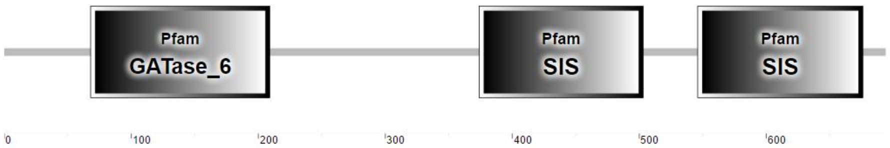

C

Species	Protein name	Per.ident	Accession
A. fumigatus A1163	Gfa1	100%	EDP49199.1
S. cerevisiae S288C	Gfa1	62.90%	NP_012818.1
C. albicans SC5314	Gfa1	64.06%	XP_721697.2
C. auris	Gfa1	63.52%	XP_028889943.2
C. neoformans	Gfa1	60.39%	XP_024514335.1
Homo sapiens	GFAT 1	55.35%	NP_001231639.1
	GFAT 2	56.21%	NP_005101.1
Mus musculus	GFAT1	55.40%	NP_038556.1
	GFAT2	56.21%	NP_038557.1

Fig. S1

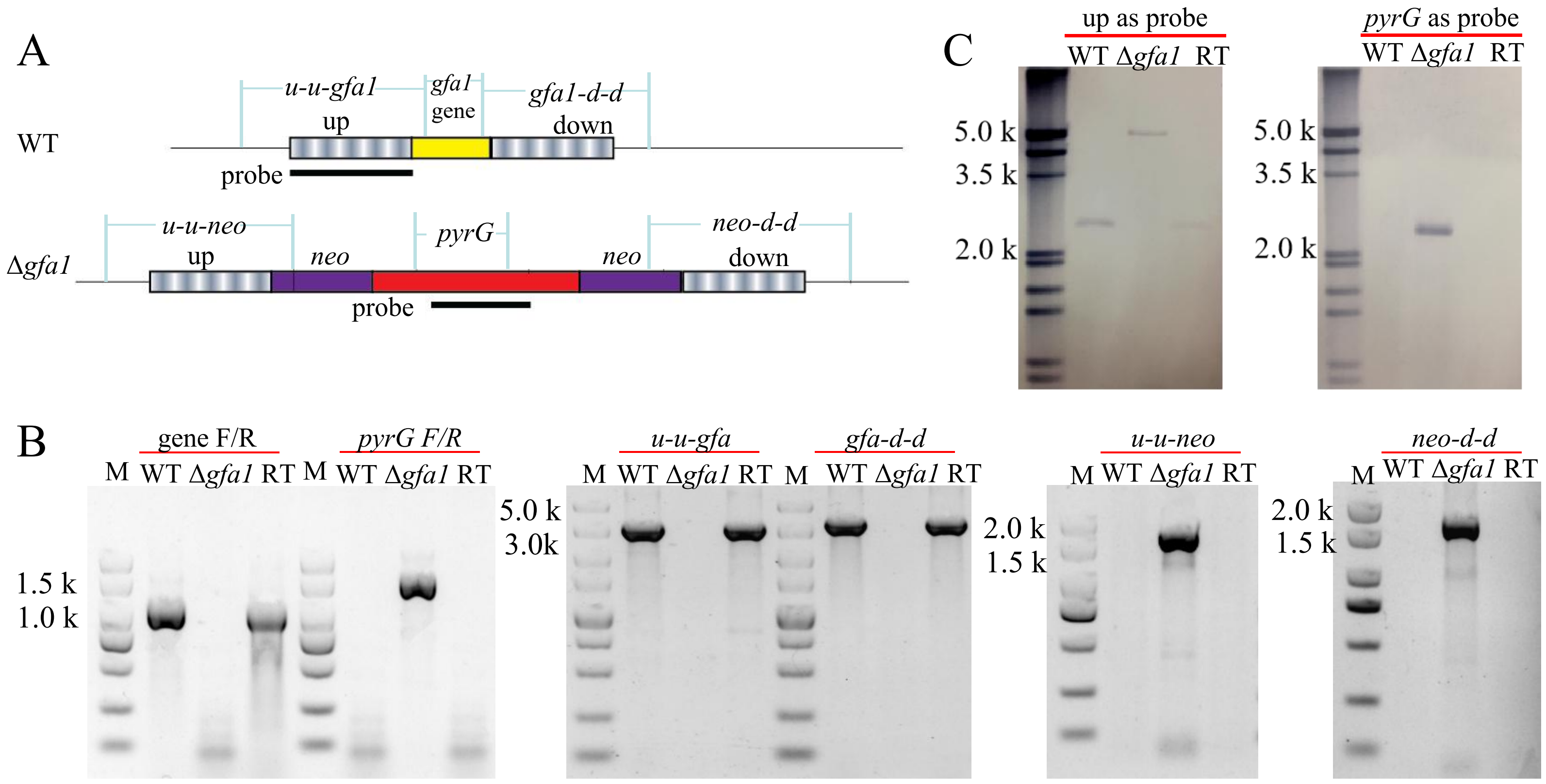
A
WT
u-u-gfa1
gfa1
gene
gfa1-d-d
up
down
probe
Δgfa1
u-u-neo
up
neo
pyrG
neo
neo-d-d
down
probe
C
up as probe
WT Δgfa1 RT
5.0 k
3.5 k
2.0 k
pyrG as probe
WT Δgfa1 RT
5.0 k
3.5 k
2.0 k
B
gene F/R
M WT Δgfa1 RT
pyrG F/R
M WT Δgfa1 RT
1.5 k
1.0 k
u-u-gfa
M WT Δgfa1 RT
gfa-d-d
M WT Δgfa1 RT
5.0 k
3.0k
u-u-neo
M WT Δgfa1 RT
2.0 k
1.5 k
neo-d-d
M WT Δgfa1 RT
2.0 k
1.5 k

Fig. S2

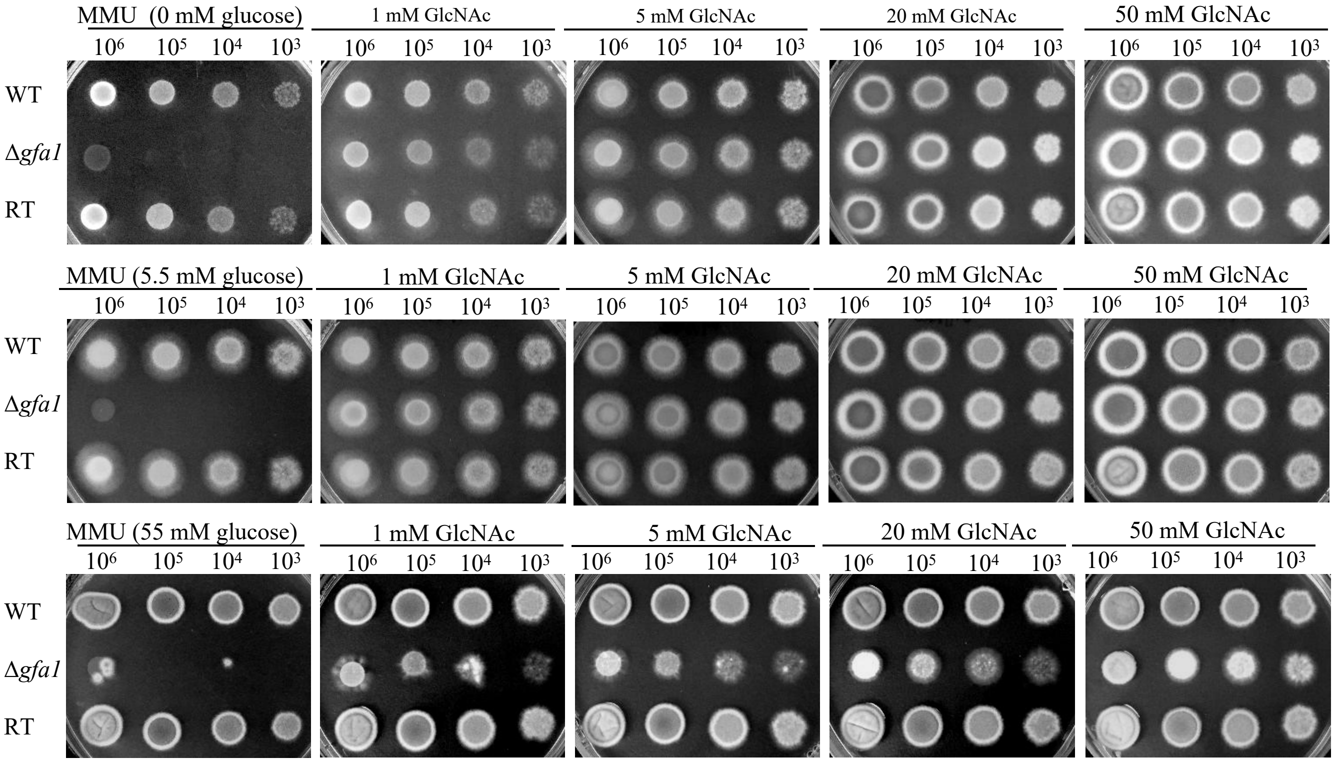
MMU (0 mM glucose)
1 mM GlcNAc
5 mM GlcNAc
20 mM GlcNAc
50 mM GlcNAc
MMU (5.5 mM glucose)
MMU (55 mM glucose)
10⁶ 10⁵ 10⁴ 10³
WT
Δgfa1
RT

Fig. S3

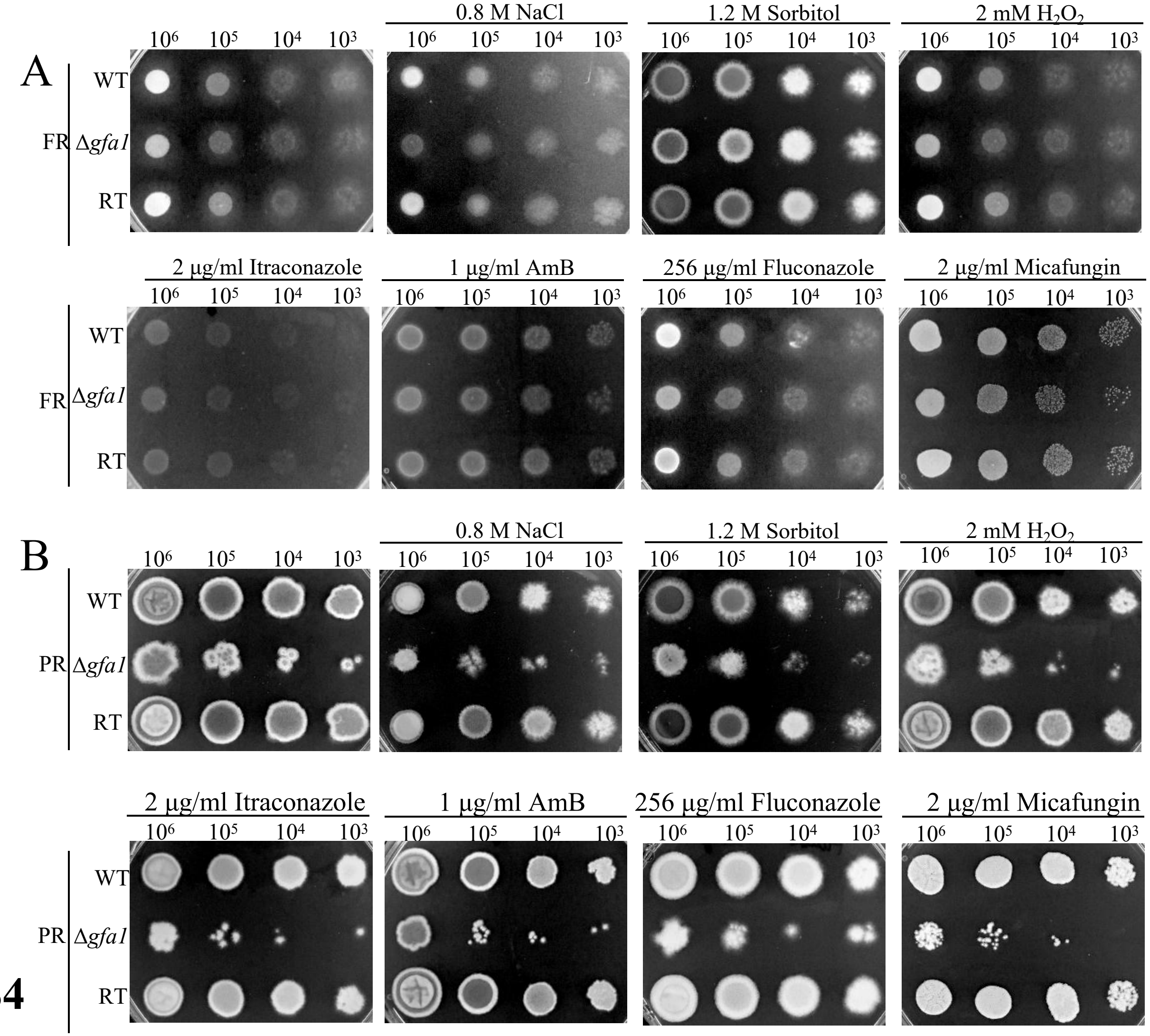
A
B
FR
PR
WT
Δgfa1
RT
10^6 10^5 10^4 10^3
0.8 M NaCl
1.2 M Sorbitol
2 mM H2O2
2 μg/ml Itraconazole
1 μg/ml AmB
256 μg/ml Fluconazole
2 μg/ml Micafungin

Fig. S4

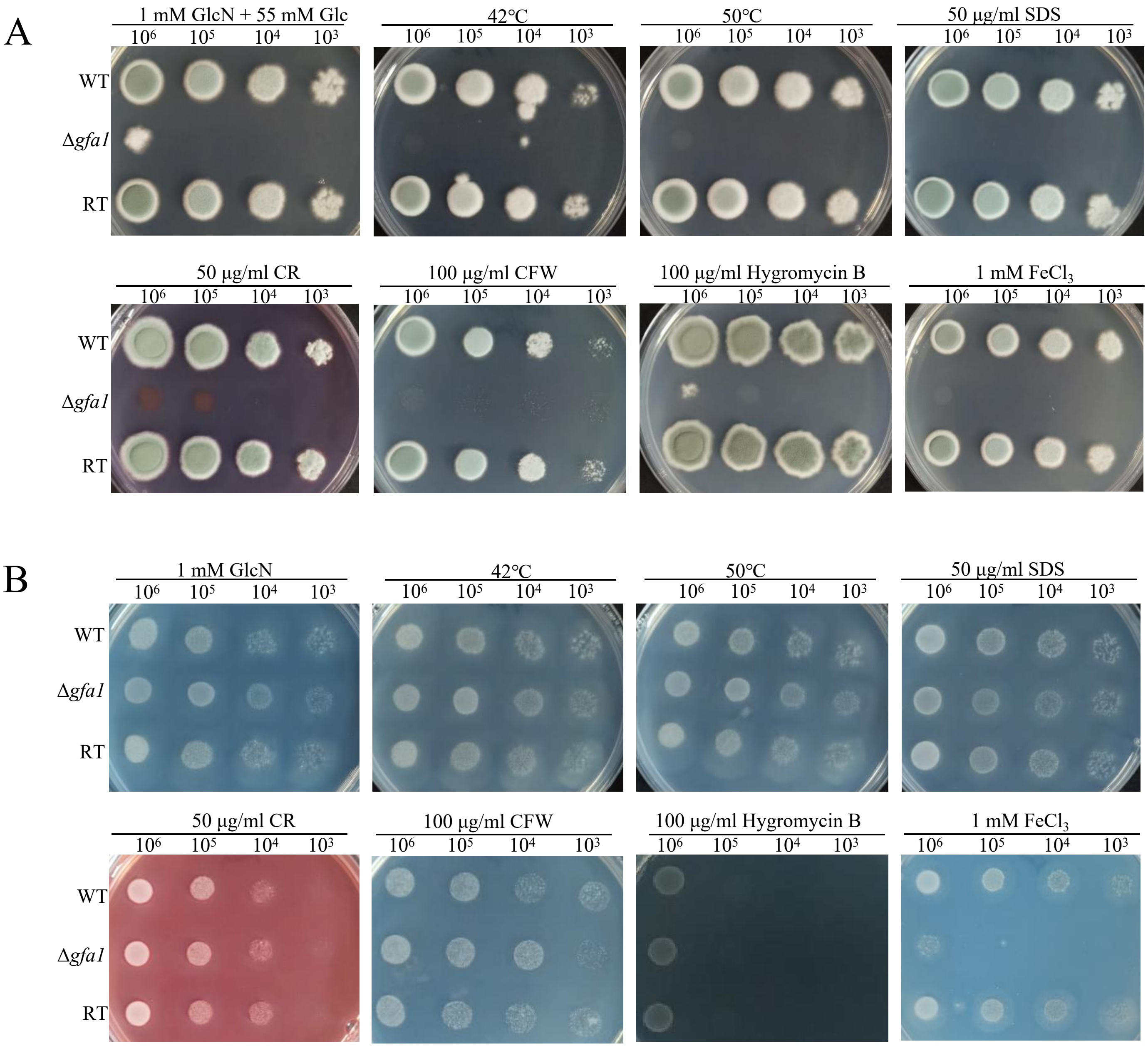
A
1 mM GlcN + 55 mM Glc
42°C
50°C
50 μg/ml SDS
50 μg/ml CR
100 μg/ml CFW
100 μg/ml Hygromycin B
1 mM FeCl3
10^6 10^5 10^4 10^3
WT
Δgfa1
RT
B
1 mM GlcN
42°C
50°C
50 μg/ml SDS
50 μg/ml CR
100 μg/ml CFW
100 μg/ml Hygromycin B
1 mM FeCl3
10^6 10^5 10^4 10^3
WT
Δgfa1
RT

Fig. S5
